# Enhancing data visualisation to capture the simulator sickness phenomenon: On the usefulness of radar charts

**DOI:** 10.1016/j.dib.2017.05.051

**Published:** 2017-05-31

**Authors:** Romain Chaumillon, Thomas Romeas, Charles Paillard, Delphine Bernardin, Guillaume Giraudet, Jean-François Bouchard, Jocelyn Faubert

**Affiliations:** a Visual Psychophysics and Perception Laboratory, School of Optometry, Université de Montréal, 3744 Jean-Brillant, Montréal, Quebec, Canada, H3T 1P1; b Essilor Canada Ltd., Montréal, Quebec, Canada; c Neuropharmacology Laboratory, School of Optometry, Université de Montréal, 3744 Jean-Brillant, Montreal, Quebec, Canada, H3T 1P1

**Keywords:** Simulator sickness, Radar charts, Driving

## Abstract

The data presented in this article are related to the research article entitled “*The use of transdermal scopolamine to solve methodological issues raised by gender differences in susceptibility to simulator sickness*” (Chaumillon et al., 2017) [1]. In an outstanding first demonstration, Kennedy et al. [2] showed that the Simulator Sickness Questionnaire (SSQ) is an appropriate tool to suit the purposes of characterizing motion sickness experienced in virtual environments. This questionnaire has since been used in many scientific studies. Recently, Balk et al. [3] suggested that the proposed segregation of SSQ scores into three subclasses of symptoms might limit the accuracy of simulator sickness assessment. These authors performed a factor analysis based on SSQ scores obtained from nine studies on driving simulators. Although their factor analysis resulted in the same three orthogonal classes of symptoms as Kennedy et al. [2], unlike this pioneering study, no items were attributed to more than one factor and five items were not attributed to any class of symptoms. As a result, they claimed that an exploration of each item score should give additional cues on individual profiles. To gain a better characterization of such item-by-item exploration, data utilised in this research are shown using a radar chart visualisation.

## **Specifications Table**

**Table:** 

Subject area	*Psychology*
More specific subject area	*Assessment of Simulator Sickness during driving*
Type of data	*Table and Figures*
How data was acquired	*Survey: simulator sickness questionnaire*
Data format	*Analysed*
Experimental factors	*Simulator sickness questionnaires were filled before and after the virtual reality immersion to investigate the influence of a high-fidelity car driving simulator and transdermal scopolamine in susceptibility to simulator sickness*
Experimental features	*Data has been configured in radar chart to gain a better understanding of fluctuations in susceptibility to simulator sickness*
Data source location	*Visual Psychophysics and Perception Laboratory, University of Montreal, Canada*
Data accessibility	*The data are available within this article* (Table 1)

**Value of the data**

• Performing an item-by-item analysis helps to better characterize which specific symptoms involved in simulator sickness are the most prominent in each population/condition. This profiling can be used to personalize solutions to reduce feelings of discomfort. The authors present these data in order to suggest that the restriction of analysis to total SSQ scores or to the three subclasses of symptoms might not be representative of the whole phenomenon of simulator sickness.
• The use of a radar chart visualisation shows, in one straightforward and clear picture, which symptoms and to what extent these symptoms are implied in the genesis of the simulator sickness. Such a methodology might be a useful tool to simplify comparisons between results obtained in further studies.
• Using this method of assessing the increasing item score between two subsequent driving sessions highlights the relevance of the methodology used to evaluate the relationship between time and simulator sickness intensity.

## 1 Data

**Fig. 1:**
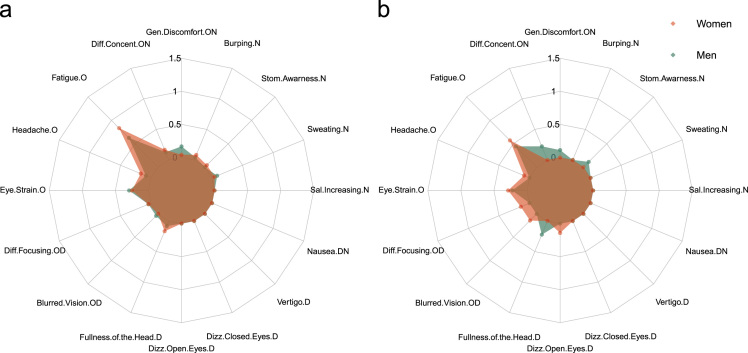
Mean scores observed in each item of the pre-exposure simulator sickness questionnaire (SSQ0). (a) Scores computed on each item of the SSQ0 during the experiment 1 for women (red area) and for men (green area). (b) Scores computed on each item of the SSQ0 in the scopolamine condition of the experiment 2 [1]. The O, D and N letters following the name of each item indicate in which class(es) of symptoms the corresponding item was involved [2]: O corresponds to Oculomotor discomfort, D to Disorientation and N to Nausea.

**Fig. 2:**
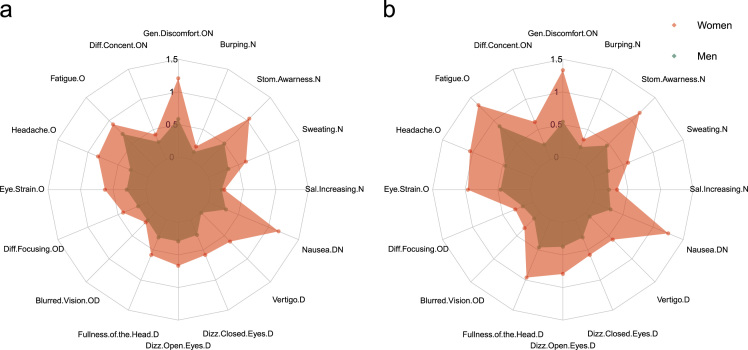
Mean scores observed in each item of the simulator sickness questionnaire during experiment 1. (a) Women (red area) and men (green area) item scores reported after the first driving session (*i.e.* SSQ1) and (b) after the second driving session (*i.e.* SSQ2). The O, D and N letters following the name of each item indicate in which class(es) of symptoms the corresponding item was involved [2]: O corresponds to Oculomotor discomfort, D to Disorientation and N to Nausea.

Initial scores obtained during a pre-exposition simulator sickness questionnaire (SSQ0; Fig. 1) as well as scores obtained in each item of the SSQ following the first (SSQ1; Fig. 2a) and the second exposition to the high-fidelity motion-based driving simulator (SSQ2; Fig. 2b) are reported using a radar chart view (see Table 1 for original dataset).

**Table 1:** Mean scores computed for each item of the simulator sickness questionnaire (SSQ). For each of the four questionnaires (*i.e.* SSQ0, SSQ1 and SSQ2 during the experiment 1 as well as SSQO during the experiment 2), mean scores were separately computed for women (W) and men (M). The O, D and N letters in the symptom class column indicate in which class(es) of symptoms the corresponding item was classified [2]: O corresponds to Oculomotor discomfort, D to Disorientation and N to Nausea.

**SSQ item**	**Symptom class**	**SSQ0 Ex 1**		**SSQ0 Ex 2**		**SSQ1 Ex 1**		**SSQ2 Ex 1**	
		**W**	**M**	**W**	**M**	**W**	**M**	**W**	**M**
*General Discomfort*	O-N	.04	.17	0	.11	1.21	.58	1.33	.54
*Fatigue*	O	.83	.63	.57	.44	.92	.71	1.33	.88
*Headache*	O	.17	.08	.09	0	.83	.29	1.04	.46
*Eyestrain*	O	.25	.29	.29	.22	.63	.29	.96	.46
*Difficulty Focusing*	O-D	.04	.04	.14	0	.42	.17	.29	.17
*Salivation Increasing*	N	0	0	0	0	.21	.17	.33	.21
*Sweating*	N	.04	.08	0	0	.63	.33	.58	.25
*Nausea*	D-N	0	0	0	0	1.17	.29	1.25	.29
*Difficulty Concentrating*	O-N	.17	.13	0	.22	.42	.29	.63	.25
*Fullness of the head*	D	.17	.08	0	.22	.58	.29	.96	.46
*Blurred Vision*	O-D	0	.04	.14	0	.21	.17	.33	.13
*Dizziness with eyes open*	D	0	0	0	0	.67	.29	.79	.38
*Dizziness with eyes closed*	D	0	0	0	0	.58	.25	.58	.29
*Vertigo*	D	0	0	0	0	.63	0	.58	.08
*Stomach Awareness*	N	.04	0	0	.11	1.04	.5	1.17	.46
*Burping*	N	.08	.04	0	0	.22	.13	.33	.21

## 2 Experimental design, materials and methods

An experiment was conducted to assess the efficiency of one technological (the use of high-fidelity motion-based driving simulator; **Experiment** 1 involving 48 participants) and one pharmacological solution (the use of transdermal scopolamine; **Experiment 2** involving 16 participants) to solve the methodological issues raised by gender differences in susceptibility to simulator sickness (see Chaumillon et al. [1]). To control the relationship between time and simulator sickness severity highlighted by previous studies [4], [5], participants were exposed to two driving sessions each lasting approximately 16 min. The driving sessions were performed in a VS500M car driving simulator (Virage Simulation Inc. ®). Participants were seated in a high-fidelity, motion-based, driving simulator which faithfully reproduced the controls and indicators that are found on the steering wheel as well as the dashboard and pedals of a vehicle interior.

Before the first exposition and after each of the two expositions, participants filled a simulator sickness questionnaire ([1]; *i.e.* SSQ0, SSQ1 and SSQ2, respectively; cf. Data in Table 1). To assess the influence of our high-fidelity, motion-based driving simulator (**Experiment 1**) and of the use of transdermal scopolamine (**Experiment 2**), on simulator sickness intensity, SSQ scores reported before and after each driving sessions were compared. Following the methodology proposed by Kennedy et al. [2], we analysed the data considering total SSQ scores and scores obtained in each of the three subclasses of symptoms (*i.e.* Oculomotor symptoms, Disorientation and Nausea). Nevertheless, a reproduction of the initial study from Kennedy et al. [2] recently demonstrated that it may be better to attend to individual item score elevation rather than overall SSQ scores [3]. To improve the characterization of the simulator sickness phenomenon, a visualisation of the data proposed here was carried out with the package “radarchart” using the software R (R development Core Team, 2008). We reported data obtained in pre-exposition questionnaires in Experiment 1 (Fig. 1**a**) and Experiment 2 (Fig. 1**b**) as well as the evolution of scores across time during Experiment 1 (Fig. 2).
